# Smoking Cessation Increases Short-Term Risk of Type 2 Diabetes Irrespective of Weight Gain: The Japan Public Health Center-Based Prospective Study

**DOI:** 10.1371/journal.pone.0017061

**Published:** 2012-02-13

**Authors:** Shino Oba, Mitsuhiko Noda, Kayo Waki, Akiko Nanri, Masayuki Kato, Yoshihiko Takahashi, Kalpana Poudel-Tandukar, Yumi Matsushita, Manami Inoue, Tetsuya Mizoue, Shoichiro Tsugane, for the Japan Public Health Center-based Prospective Study Group

**Affiliations:** 1 Department of Health Promotion, National Institute of Public Health, Saitama, Japan; 2 Department of Diabetes and Metabolic Medicine, Diabetes Research Center, National Center for Global Health and Medicine, Tokyo, Japan; 3 Department of Ubiquitous Health Informatics, The University of Tokyo Hospital, Tokyo, Japan; 4 Department of Diabetes and Metabolic Diseases, Graduate School of Medicine, The University of Tokyo, Tokyo, Japan; 5 Department of Epidemiology and Prevention, International Clinical Research Center, National Center for Global Health and Medicine, Tokyo, Japan; 6 Japan Foundation for the Promotion of International Medical Research Cooperation, Tokyo, Japan; 7 Waseda Institute for Advanced Study, Waseda University, Tokyo, Japan; 8 Department of Clinical Research Coordination, International Clinical Research Center, National Center for Global Health and Medicine, Tokyo, Japan; 9 Epidemiology and Prevention Division, Research Center for Cancer Prevention and Screening, National Cancer Center, Tokyo, Japan; University of Padova, Medical School, Italy

## Abstract

**Objective:**

The effect of smoking cessation on the risk of diabetes has been reported previously. However, it is unknown whether the association is influenced by weight gain and other potential risk factors.

**Methods:**

The Japan Public Health Center-Based Prospective Study established in 1990 for Cohort I and in 1993 for Cohort II provided data, and 25,875 men and 33,959 women were analyzed. The response rate to the baseline questionnaire was 80.9%, and 68.4% of the respondents participated both the 5- and 10-year follow-up surveys. Smoking cessation was noted during the initial five years and the development of diabetes was reported in the subsequent five years.

**Results:**

An increased risk was observed among individuals who newly quit smoking compared with never smokers among men (odds ratio (OR) = 1.42, 95% CI = 1.03–1.94) and women (OR = 2.84, CI = 1.53–5.29). The risk of developing diabetes among male new quitters who gained 3 kg or more during the 5-year follow-up did not substantially differ from the risk among male never smokers with less than 3 kg of weight gain or no weight gain, while an increased risk was observed among male new quitters with less or no weight gain (OR = 1.46, 95%CI 1.00–2.14). An insignificant increased risk was observed among male new quitters with a family history of diabetes compared with male never smokers with a family history of diabetes. The risk was more than twice as high for male new quitters who used to smoke 25 or more cigarettes per day compared with never smokers (OR = 2.15, 95%CI: 1.34–3.47).

**Discussion:**

An increased risk of diabetes was implied among individuals who quit smoking. However, the increased risk was not implied among those who gained weight over the 5-years of follow-up. Those who had major risk factors for diabetes or who smoked heavier had a higher risk.

## Introduction

A few prospective studies have assessed the impact of smoking cessation on the development of diabetes, and they have reported that smoking cessation increased the subsequent risk of diabetes [1], [2], [3]. Weight gain after smoking cessation and related physiological changes have been suggested as possible causes of this adverse effect of smoking cessation, although an increased risk was observed even after controlling for the weight change. However, a larger number of subjects, including subjects who stop smoking during the follow-up period, is needed for further assessments of the effects of weight gain and other potential risk factors for diabetes.

In Japan, the prevalence of smoking among men has steadily decreased over time. The smoking prevalence among men was 59.7% in 1986 and 39.4% in 2007 [4], [5]. There is an important public health interest in evaluating the health effects of smoking cessation in this country. With the increasing trend of smoking cessation especially among male smokers in Japan, we expected a relatively large number of subjects who stopped smoking within the cohort, which enabling us to further evaluate the effects of potential risk factors for diabetes on the association between smoking cessation and the subsequent risk of diabetes.

The current study was conducted to evaluate the association between smoking cessation and the risk of the development of diabetes prospectively among men and women in a Japanese population. The study also aimed to identify a high-risk group among individuals who stopped smoking by evaluating the 5-year weight gain, potential risk factors, and the frequency and duration of cigarette smoking. The duration of time since smoking cessation among former smokers was also evaluated.

## Methods

### Study population

The Japan Public Health Center-Based Prospective Study (JPHC) was initiated in 1990 for Cohort I, and Cohort II was added in 1993. The study design has been previously described in detail [6]. Briefly, subjects were from eleven public health center areas across Japan, and the study population was the residents of each area who were 40 to 69 years old at the time of the baseline survey. A questionnaire was administered at baseline, at which time the participants were informed of the objectives of the study. Those who responded to the questionnaire were regarded as having consented to participate in the study. Follow-up surveys were conducted at five and ten years after the baseline survey. The study was approved by the Institutional Review Board of the National Cancer Center, Tokyo, Japan.

**Figure 1:**
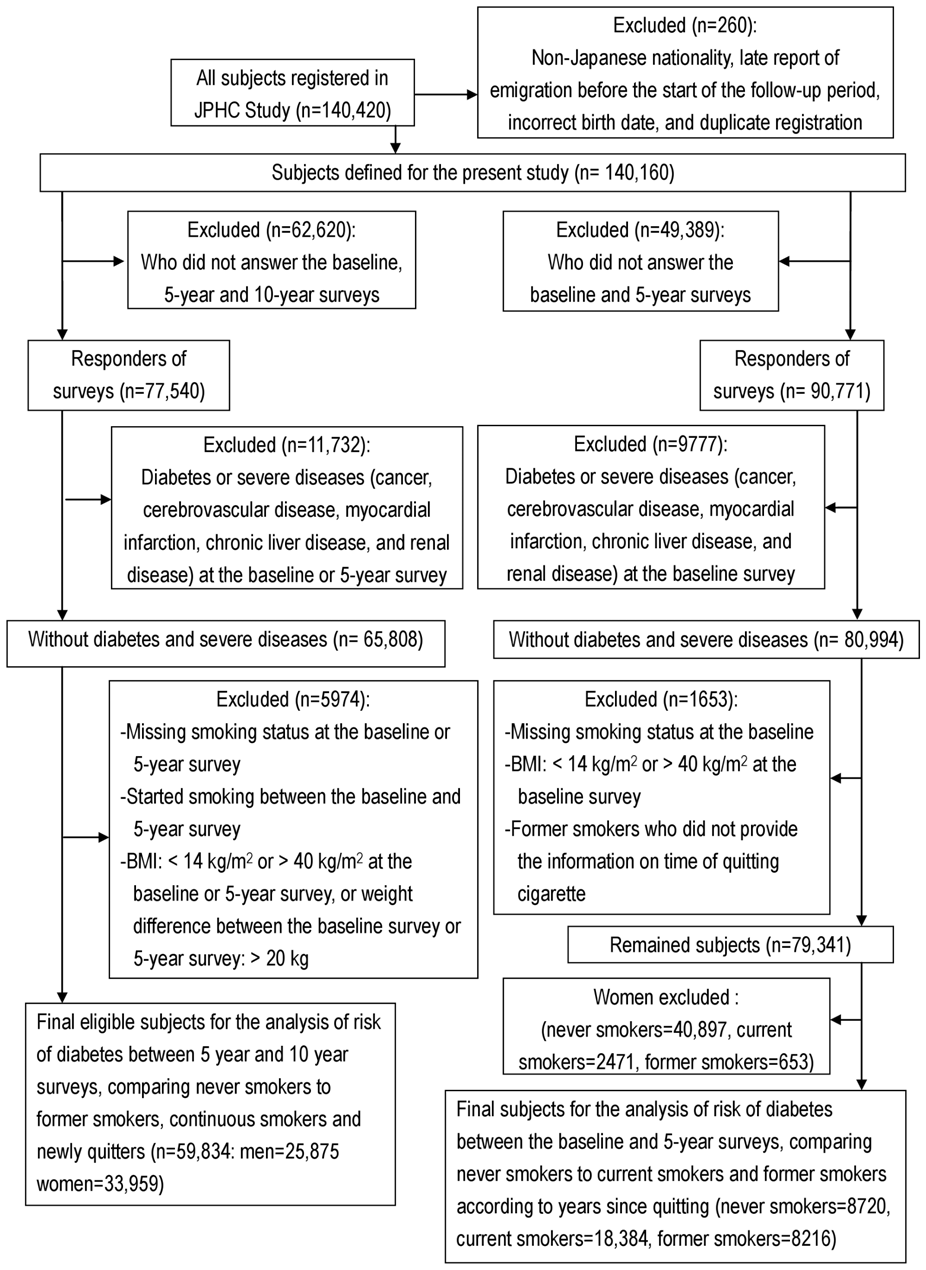
Flow of study population.

Of the 140,160 eligible subjects, 113,403 responded to the questionnaire at the time of the baseline survey, yielding a response rate of 80.9%. Of the respondents, 77,540 (68.4%) responded to both the 5- and 10-year follow-up surveys. Those who reported having been diagnosed as having diabetes at the 5-year survey period, those who reported a history of cancer, cerebrovascular disease, myocardial infarction, chronic liver disease, or renal disease at baseline, those whose body mass index (BMI) at baseline or at the time of the 5-year survey was either less than 14 kg/m^2^ or equals to or larger than 40 kg/m^2^, those whose weight or height information was missing from the baseline or 5-year survey, and those whose weight change between the baseline and 5-year surveys exceeded 20 kg were excluded from the analysis. Since the current study focused on smoking cessation, 1247 participants who started cigarette smoking between the baseline and 5-year surveys were also excluded. After these exclusions, the analysis included 59,834 eligible participants consisting of 25,875 men and 33,959 women (Figure 1). The analysis to assess the effect of weight gain and to attempt to identify high-risk groups was conducted among male participants, as a sufficient number of new quitters was only obtained among the men.

### Smoking status

Smoking status was identified from the questionnaires administered at the baseline and 5-year surveys. Using this information, participants were classified into never smokers, former smokers (at baseline), continuous smokers (who were current smokers in both surveys), and new quitters (who quit smoking between the baseline survey and the 5-year survey). Those who reported themselves as current smokers at the baseline survey and then reported themselves as never-smokers at the 5-year survey were classified as new quitters (n = 505 for men and n = 242 for women). Likewise, those who reported themselves as former smokers at the baseline survey and then reported themselves as never-smokers at the 5-year survey were classified as former smokers (n = 2777 for men and n = 292 for women). The number of cigarettes smoked per day was asked in the questionnaire, and the number of years of smoking was also estimated from the answers in the questionnaires. For new quitters, as information on the exact timing of smoking cessation during the 5-year follow-up was not reported, the number of years of smoking was estimated by adding 2.5 to the years of smoking at the baseline survey. The pack-year history was calculated based on the number of packs of cigarettes smoked per day multiplied by the number of years of smoking.

### Other characteristics of the participants

Weight, height, and the family history of diabetes were recorded using the baseline questionnaire. BMI (body mass index) was calculated as the weight in kilograms divided by the square of the height in meters. The self-reported BMI in the JPHC study has been previously validated [7]. The self-reported BMIs (mean: 23.45 kg/m^2^ in men and 23.57 kg/m^2^ in women) were slightly lower than the measured BMIs (mean: 23.54 kg/m^2^ in men and 23.78 kg/m^2^ in women), and the Spearman correlation coefficients were 0.89 in men and 0.91 in women. A diagnosed history of hypertension was reported in the 5-year survey. Alcohol intake at the time of the 5-year survey was estimated using a validated food frequency questionnaire [8]. The family history of diabetes was reported in the questionnaire in the baseline survey. The weight change between the baseline survey and the 5-year survey was calculated. Total physical activity level and leisure time physical activity were estimated from the questionnaire in the 5-year survey. The total physical activity level was transferred into metabolic equivalents per day, a previously validated measure [9].

### Ascertainment of the development of diabetes

The development of newly diagnosed diabetes between the 5-year survey and the 10-year survey was identified using the questionnaires. The validity of self-reported diabetes was assessed previously for study participants in three districts of the study areas. Among the participants whose medical records were available, 94% of the participants who reported a diagnosis of diabetes were confirmed to have diabetes based on their medical records [10].

### Statistical analysis

The cumulative incidence for the development of diabetes during between the 5-year survey and 10-year survey was computed according to smoking status and sex. A logistic regression analysis was used to assess whether former smokers, continuous smokers or new quitters were more likely to develop diabetes than never smokers. The analysis was adjusted for age and study area and further adjusted for BMI at the time of the 5-year survey, history of hypertension, alcohol intake, family history of diabetes, and weight change between the baseline and 5-year surveys. Leisure-time physical activity was also added to the model, as it appeared to vary according to smoking status.

To explore the influence of the 5-year weight gain and to identify a high-risk group among new quitters compared with never smokers, separate models were run. Only male participants were analyzed, as the number of female new quitters was insufficient. Dichotomous classifications of the following variables were considered: weight gain of 3 kg, BMI of 23 kg/m^2^, alcohol consumption on one day per week, median level of physical activity in the 5-year survey, and family history of diabetes. This cut-off point for the BMI was based on the criteria of the regional office for the Western Pacific Region of WHO, which was reported to be suitable for Japanese subjects [11]. Analyses categorizing new quitters according to the number of cigarettes per day, the duration of smoking, and the pack-year history were also conducted, and the p values for trends were calculated by treating the median value in each category as a continuous value.

We conducted a separate analysis to evaluate the association between time since smoking cessation and the subsequent risk of diabetes. As information for the timing of smoking cessation among former smokers was available for the baseline survey but not for the 5-year survey, we examined the data obtained from the baseline survey and calculated the duration since they had quit smoking cigarettes. The development of diabetes between the baseline survey and 5-year survey was evaluated among former smokers categorized according to the time that had passed since quitting cigarettes and compared with the development among the never smokers. The risk among current smokers was also evaluated at the same time in the model. Only male participants were analyzed, as the number of female participants was insufficient. Details on the inclusion in or exclusion from the analysis are given in Figure 1. Never smokers comprised the reference group, and the association between the smoking categories and the risk of diabetes was assessed in a logistic regression model. We first adjusted for age and the study area; then, adjustments for the history of hypertension, alcohol intake at the baseline survey, family history of diabetes, and leisure time physical activity at the baseline survey were added to the model; and the BMI at the time of the baseline survey was added to a third model. All the statistical analyses were performed with SAS software (SAS Institute Inc, Cary, NC).

## Results

Tables 1 and 2 summarize the characteristics of the participants at baseline and at the time of the 5-year survey according to smoking status. During the follow-up between the 5-year and 10-year surveys, 653 men (2.52%) and 447 women (1.32%) reported that they had been newly diagnosed as having diabetes. Table 3 summarizes the association between smoking status and the risk for the development of diabetes. Among men, continuous smokers and new quitters had a significantly increased risk of diabetes after controlling for weight changes and other potential risk factors for diabetes. Among women, a significantly increased risk of diabetes was observed among former smokers, continuous smokers and new quitters, compared with never smokers, in a multivariate analysis, and the risk was highest among new quitters.

**Table 1:** Characteristics of 25,875 Men According to Smoking Status in Baseline and 5-Year Surveys in the Japan Public Health Center-Based Prospective Study.

	Overall	Never smokers	Former smokers	Continuous smokers	New quitters^2^
n	25,875	6,265	5,913	11,813	1,884
**Demographic characteristics**					
Mean Age at 5-year survey (SD)	56.3 (7.7)	56.4 (7.2)	57.9 (8.0)	55.1 (7.4)	57.5 (8.0)
Family history of diabetes (%)	8.3	8.3	7.2	9.0	7.4
**Behavioral characteristics**					
Alcohol consumption (%)					
≥1 day per week	68.5	62.8	71.0	71.3	62.3
Physical activity level (METS/day)^1^	33.9 (6.7)	34.1 (6.8)	33.7 (6.7)	34.0 (6.8)	33.5 (6.7)
Leisure-time physical activity (%)					
≥1 day per week	20.8	23.6	25.8	16.7	20.7
**Clinical variables**					
History of hypertension (%)	16.7	17.2	21.8	13.2	20.2
**Weight related variables**					
Mean weight at baseline (SD), kg	63.2 (8.6)	63.8 (8.5)	63.9 (8.5)	62.6 (8.6)	63.0 (8.7)
Mean weight at 5-year survey (SD), kg	63.5 (8.8)	64.1 (8.7)	64.1 (8.6)	62.7 (8.9)	64.3 (9.1)
Mean weight change (SD), kg	0.3 (3.6)	0.3 (3.5)	0.2 (3.5)	0.2 (3.6)	1.3 (4.3)
Mean weight change % of baseline weight	0.6	0.6	0.4	0.4	2.2
Weight change > = 3 kg (%)	20.9	20.0	19.7	19.6	35.5
Mean BMI at baseline (SD), kg/m^2^	23.4 (2.7)	23.9 (2.7)	23.8 (2.7)	23.0 (2.7)	23.3 (2.7)
Mean BMI at 5-year survey (SD), kg/m^2^	23.5 (2.8)	24.0 (2.8)	23.8 (2.7)	23.1 (2.8)	23.8 (2.9)
Mean change of BMI (SD), kg/m^2^	0.1 (1.5)	0.1 (1.5)	0.1 (1.5)	0.1 (1.5)	0.5 (1.8)
Mean BMI change % of baseline BMI	0.6	0.6	0.5	0.4	2.3
**Smoking related variables**					
Mean intensity of smoking, cigarettes/day			23.9 (14.0)	22.5 (11.4)	19.5 (11.2)
Mean duration of smoking, years reported			21.3 (10.2)	35.1 (7.7)	32.7 (9.3)
Mean pack-Year			26.9 (21.5)	38.8 (20.2)	32.0 (19.5)
Mean years since quitting^1^			13.0 (8.4)		

1 The numbers differ as a result of missing data for physical activity level for which it was 21,553, and years since quitting of former smokers for which it was 5871.

2 Who stopped smoking between the baseline and 5-year surveys.

**Table 2:** Characteristics of 33,959 Women According to Smoking Status in Baseline and 5-Year Surveys in the Japan Public Health Center-Based Prospective Study.

	Overall	Never smokers	Former smokers	Continuous smokers	New quitters^2^
n	33,959	31,866	412	1,323	358
**Demographic characteristics**					
Mean Age at 5-year survey (SD)	56.5 (7.7)	56.6 (7.7)	55.3 (8.5)	54.2 (7.2)	55.0 (7.0)
Family history of diabetes (%)	8.8	8.7	14.1	10.6	8.9
**Behavioral characteristics**					
Alcohol consumption (%)					
≥1 day per week	12.7	11.3	32.8	36.1	23.7
Physical activity level (METS/day)^1^	32.9 (5.7)	32.8 (5.7)	32.5 (5.2)	33.3 (5.9)	33.0 (5.3)
Leisure-time physical activity (%)					
≥1 day per week	20.7	20.7	27.4	18.5	18.7
**Clinical variables**					
History of hypertension (%)	17.8	18.1	18.7	12.1	14.8
**Weight related variables**					
Mean weight at baseline (SD), kg	53.9 (7.4)	53.9 (7.4)	54.6 (7.7)	53.8 (8.1)	53.6 (7.8)
Mean weight at 5-year survey (SD), kg	54.1 (7.6)	54.1 (7.6)	54.7 (7.8)	53.8 (8.3)	54.0 (7.9)
Mean weight change (SD), kg	0.2 (3.3)	0.2 (3.3)	0.1 (3.7)	0.02 (3.6)	0.4 (4.3)
Mean weight change % of baseline weight	0.4	0.4	0.4	0.2	1.1
Weight change > = 3 kg (%)	17.9	17.8	19.2	18.3	25.7
Mean BMI at baseline (SD), kg/m^2^	23.3 (3.3)	23.4 (3.0)	23.4 (3.3)	22.8 (3.3)	23.0 (3.3)
Mean BMI at 5-year survey (SD), kg/m^2^	23.4 (3.1)	23.5 (3.1)	23.4 (3.2)	22.8 (3.3)	23.2 (3.2)
Mean change of BMI (SD), kg/m^2^	0.1 (1.6)	0.1 (1.6)	0.1 (1.9)	0.02 (1.7)	0.2 (2.1)
Mean BMI change % of baseline BMI	0.6	0.7	0.6	0.4	1.4
**Smoking related variables**					
Mean intensity of smoking, cigarettes/day			10.9 (8.3)	15.6 (9.6)	10.0 (6.8)
Mean duration, years reported			13.3 (9.3)	26.7 (8.5)	20.2 (10.4)
Mean pack-Year			8.4 (10.5)	21.0 (14.5)	11.2 (11.5)
Mean years since quitting^1^			11.3 (7.8)		

1 The numbers differ as a result of missing data for physical activity level for which it was 28,370, and years since quitting of former smokers for which it was 399.

2 Who stopped smoking between the baseline and 5-year surveys.

**Table 3:** Risk Estimates for Development of Diabetes According to Status of Cigarette Smoking During Follow-Up Between 5-Year Survey and 10-Year Survey in the Japan Public Health Center-Based Prospective Study.

Smoking Exposure	No. of developed cases	n	% of developed cases	Age and area adjusted OR (95% CI)	Multivariate^2^OR (95% CI)
*Men*					
Smoking status (n = 25,875)					
Never smokers	144	6265	2.3%	1.00	1.00
Former smokers	134	5913	2.3%	0.99 (0.78–1.25)	0.98 (0.77–1.25)
Continuous smokers	318	11813	2.7%	1.22 (1.00–1.50)	1.43 (1.16–1.76)
New quitters^1^	57	1884	3.0%	1.34 (0.98–1.83)	1.42 (1.03–1.94)
*Women*					
Smoking status (n = 33,959)					
Never smokers	404	31866	1.3%	1.00	1.00
Former smokers	10	412	2.4%	2.17 (1.15–4.11)	2.16 (1.13–4.13)
Continuous smokers	22	1323	1.7%	1.48 (0.96–2.30)	1.68 (1.07–2.63)
New quitters^1^	11	358	3.1%	2.62 (1.42–4.81)	2.84 (1.53–5.29)

1 Who stopped smoking between the baseline and 5-year surveys.

2 Multivariate adjustment: age, BMI, history of hypertension, alcohol intake, family history of diabetes, weight change between baseline and 5-year surveys, study area, leisure-time physical activity.

The associations between smoking status and the risk of diabetes according to dichotomously categorized groups for weight gain and known risk factors are presented in Table 4. Compared with never smokers whose weight gain was less than 3 kg, the equivalent counterparts among new quitters were significantly more likely to develop diabetes. In contrast, new quitters who gained 3 kg or more did not have an increased risk. The risk was close to unity among never smokers with a weight gain of 3 kg or more, and an insignificantly decreased risk was observed among them after adjustments for other predefined risk factors. The initial BMI (at the time of the 5-year survey) was the main contributor to the difference (data not shown). We conduct an additional analysis with 5 kg cut-off point for weight gain, and the results were not altered: multivariate ORs were 0.63 (0.33–1.19) for never smokers with weight gain, 1.36 (0.95–1.93) for new quitters without weight gain and 1.00 (0.54–1.84) for new quitters with weight gain compared to the never smokers without weight increase. Compared with the never smokers whose initial BMI was less than 23, all the other groups had higher risks of diabetes, and the risk was about three times higher among new quitters with a higher BMI. New quitters whose total physical activity levels were lower than the median level had a non-significant increased risk, whereas those with a higher level did not have an increased risk of diabetes, compared with the never smokers whose total physical activity level was less than the median level. New quitters who consumed alcohol less than once a week were more likely to develop diabetes, compared with their never smoking counterparts. New quitters with a family history of diabetes were insignificantly more likely to develop diabetes compared with the never smokers with a family history of diabetes. We conducted an additional analysis stratified by family history of diabetes. Among 657 never smokers and new quitters with a family history of diabetes, the multivariate OR of diabetes for new quitters compared to never smokers was 1.23 (0.47–3.17). Among 7492 never smokers and new quitters without any family history of diabetes, the multivariate OR of diabetes for new quitters compared to never smokers was 1.41 (1.00–2.00).

**Table 4:** Risk Estimates for Development of Diabetes According to Weight Gain and Known Risk Factors Among Never Smoking Men and Those who Newly Quit Smoking (Between Baseline and 5-Year Surveys) in the Japan Public Health Center-Based Study, Between 5-Year Survey and 10-Year Survey.

Smoking Exposure	n	% of developed cases	Age and area adjusted OR (95% confidence interval)	Multivariate^7^ OR (95% confidence interval)
Weight gain by 3 kg (n = 8149)^1^				
Never smokers, weight gain <3 kg	5012	2.3%	1.00	1.00
Never smokers, weight gain ≥3 kg	1253	2.3%	1.03 (0.68–1.56)	0.79 (0.51–1.20)
New quitters^2^, weight gain <3 kg	1216	3.1%	1.41 (0.97–2.06)	1.46 (1.00–2.14)
New quitters, weight gain ≥3 kg	668	2.8%	1.28 (0.78–2.10)	0.97 (0.58–1.60)
BMI at the 5-year survey (n = 8149)^3^				
Never smokers, BMI<23	2275	1.3%	1.00	1.00
Never smokers, BMI≥23	3990	2.9%	2.34 (1.55–3.54)	2.38 (1.56–3.63)
New quitters, BMI<23	727	2.5%	1.98 (1.09–3.59)	1.95 (1.07–3.55)
New quitters BMI≥23	1157	3.4%	2.77 (1.70–4.52)	2.94 (1.78–4.86)
Alcohol consumption (n = 8059)^4^				
Never smokers, alcohol consumption <1 day/wk	2279	2.3%	1.00	1.00
Never smokers, alcohol consumption ≥1day/wk	3931	2.3%	1.00 (0.70–1.41)	0.99 (0.69–1.40)
New quitters, alcohol consumption <1 day/wk	675	4.3%	1.95 (1.22–3.11)	2.02 (1.26–3.24)
New quitters, alcohol consumption ≥1 day/wk	1147	2.4%	1.04 (0.65–1.67)	1.05 (0.65–1.70)
Physical activity (n = 6748)^5^				
Never smokers, METs/day<median	2730	2.3%	1.00	1.00
Never smokers,METs/day≥median	2494	2.2%	0.94 (0.65–1.37)	0.96 (0.66–1.40)
New quitters, METS/day<median	826	3.3%	1.45 (0.92–2.30)	1.53 (0.96–2.45)
New quitters,METS/day≥median	698	2.0%	0.88 (0.49–1.58)	0.97 (0.54–1.76)
Family history of diabetes (n = 8149)^6^				
Never smokers, with family history of diabetes	518	3.9%	1.00	1.00
Never smokers, without family history of diabetes	5747	2.2%	0.55 (0.34–0.89)	0.59 (0.36–0.95)
New quitters, with family history of diabetes	139	5.8%	1.59 (0.68–3.70)	1.51 (0.64–3.57)
New quitters, without family history of diabetes	1745	2.8%	0.74 (0.68–3.70)	0.81 (0.47–1.39)

1 Multivariate analysis was not adjusted for weight change.

2 Who stopped smoking between the baseline and 5-year surveys.

3 Multivariate analysis was not adjusted for BMI at 5-year follow-up.

4 Multivariate analysis was not adjusted for alcohol intake.

5 Multivariate analysis was not adjusted for physical activity level. Median value was 33.65 METs/day.

6 Multivariate analysis was not adjusted for family history of diabetes.

7 Age, BMI, history of hypertension, alcohol intake, family history of diabetes, weight change between baseline and 5-year surveys, study area, and leisure-time physical activity.

For new quitters, the risk of diabetes increased significantly with an increase in quantitative measures of cigarette smoking, which were the number of cigarettes per day, the duration of smoking, and the pack-year history of smoking (Table 5). The risk for the development of diabetes among new quitters who used to smoke 25 cigarettes per day or more was more than twice as large as the risk of never smokers. An increased risk was also observed among new quitters who had smoked cigarettes for more than 30 to 40 years. New quitters with more than 30 pack-years had a significantly higher risk of diabetes, compared with never smokers.

**Table 5:** Risk Estimates for Development of Diabetes According to Quantitative Measures of Cigarette Smoking Among Never Smoking Men and Those who Newly Quit Smoking (Between Baseline and 5-Year Surveys) in the Japan Public Health Center-Based Study, Between 5-Year Survey and 10-Year Survey.

Smoking Exposure	n	% of developed cases	Age and area adjusted OR (95% confidence interval)	Multivariate^2^ OR (95% confidence interval)
Number of cigarettes/day (n = 8149)				
Never smokers	6265	2.3%	1.00	1.00
New quitters^1^, number of cigarettes/day <15	526	1.7%	0.75 (0.38–1.48)	0.81 (0.41–1.60)
New quitters, number of cigarettes/day 15–24	907	2.9%	1.29 (0.84–1.98)	1.35 (0.87–2.09)
New quitters, number of cigarettes/day 25–	451	4.9%	2.25 (1.41–3.58)	2.15 (1.34–3.47)
p for trend			0.003	0.002
Duration of smoking, years (n = 8133)				
Never smokers	6265	2.3%	1.00	1.00
New quitters, duration of smoking <30 years	739	2.3%	1.02 (0.60–1.72)	1.05 (0.62–1.79)
duration of smoking 30<–40 years	684	4.4%	2.01 (1.33–3.02)	1.99 (1.31–3.03)
duration of smoking >40 years	445	2.2%	1.02 (0.51–2.04)	1.10 (0.54–2.24)
p for trend			0.05	0.03
Pack-Year (n = 8133)				
Never smokers	6265	2.3%	1.00	1.00
New quitters, −20 pack-year	499	1.6%	0.70 (0.34–1.43)	0.73 (0.35–1.51)
New quitters, 20<–30 pack-year	421	2.6%	1.16 (0.62–2.17)	1.32 (0.70–2.48)
New quitters, 30<–40 pack-year	431	4.2%	1.95 (1.18–3.24)	1.92 (1.14–3.23)
New quitters, 40<pack-year	517	3.9%	1.80 (1.10–2.94)	1.76 (1.07–2.90)
p for trend			0.003	0.004

1 Who stopped smoking between the baseline and 5-year surveys.

2 Age, BMI, history of hypertension, alcohol intake, family history of diabetes, weight change between baseline and 5-year surveys, study area, and leisure-time physical activity.

The 5-year risks of diabetes among former smokers according to the duration since smoking cessation, and among current smokers compared with those for never smokers are shown in Table 6. A significantly increased risk of diabetes was observed among former smokers who had quit smoking cigarettes less than 5 years ago in all the models, including the final multivariate model adjusted for BMI. However, the diabetes risk was not increased among former smokers who had quit smoking at least five years previously.

**Table 6:** Risk Estimates for Development of Diabetes Between Baseline and 5-Year Surveys According to Initial Cigarette Smoking Status and Years Since Quitting Cigarettes Among Men in the Japan Public Health Centered-Based Prospective Study.

Smoking Exposure	n of developed cases	n	% of cases	Age and area adjusted OR (95% confidence interval)	Multivariate OR^1^ (95% confidence interval)	Multivariate OR^2^ (95% confidence interval)
n = 35,320						
Never smokers	291	8720	3.3%	1.00	1.00	1.00
Current smokers	577	18384	3.1%	0.97 (0.84–1.12)	0.95 (0.82–1.10)	1.06 (0.91–1.23)
Former smokers						
<5 years since quitting	87	1888	4.6%	1.42 (1.11–1.82)	1.40 (1.09–1.79)	1.41 (1.10–1.81)
5–<10 years since quitting	77	2030	3.8%	1.15 (0.89–1.49)	1.13 (0.87–1.46)	1.12 (0.87–1.46)
10–<15 years since quitting	46	1780	2.6%	0.77 (0.56–1.05)	0.72 (0.53–1.00)	0.73 (0.53–1.00)
15–<20 years since quitting	35	1148	3.1%	0.90 (0.63–1.29)	0.86 (0.60–1.23)	0.87 (0.61–1.25)
20 - years since quitting	49	1370	3.6%	0.99 (0.72–1.35)	0.95 (0.70–1.30)	0.95 (0.69–1.29)

1 Adjusted for age, hypertension, alcohol intake, family history of diabetes, study area and leisure time physical activity.

2 Multivariate model additionally adjusted for BMI.

## Discussion

In this population-based prospective study in Japan, participants who quit smoking during the initial 5-year follow-up period had an increased risk of developing diabetes during the next five years. We also observed that new quitters with a high initial BMI, with a family history of diabetes, or who smoked heavily had a high risk of diabetes. Unlike the association with the initial BMI, the weight gain during the 5-year follow-up was not significantly associated with an increased risk among never smokers or even among new quitters. The risk did not increase among new quitters with a high physical activity level. Among former smokers who stopped smoking within the initial five years, the risk of diabetes in the following five years was higher, compared with never smokers.

The increased risk among new quitters was observed even after controlling for the weight change that occurred during the period in which smoking cessation occurred. Men and women were separately analyzed and observed the increased risk in both sexes. The increased risk of diabetes among subjects who had newly quit smoking was also observed in the previous studies, which followed smoking cessation and assessed its effects on the subsequent risk of diabetes as similar as the current study, but only among men or among men and women together [1], [2], [3]. Our study further assessed the influence of weight gain and other potential risk factors on this association.

The 5-year weight gain itself was not associated with an increased risk among new quitters. In contrast, the new quitters whose weight gain was less than 3 kg had a significant increased risk of diabetes. This finding contradicts what was discussed in the previous studies, in which weight gain following smoking cessation was suspected as a determinant of the increased risk of diabetes among new quitters [1], [2]. Since the weight gain with smoking cessation occurred in short-term, the period might not be long enough to influence on the risk of diabetes. Although numerous studies have reported that weight gain itself increases the subsequent risk of diabetes, many of them reported an association with weight gain during early adulthood or that occurred over a long term [12], [13], [14]. On the contrary, several studies, including a study conducted with the same cohort as the current study, reported that weight gain during late adulthood was less influential than weight gain during early adulthood or over the long term with regard to the risk of diabetes [15], [16], [17], [18]. However, these studies evaluated the weight change in general, and the weight gain with smoking cessation might have additional unknown factors that affect the risk of diabetes. The current finding may imply that the increased risk of diabetes among new quitters was not mediated by weight gain.

The risk of diabetes increased among former smokers who quit smoking within five years, whereas the risk was moderated among those who quit smoking five or more years ago. This result is generally consistent with the results of previous cohort studies, although a small increased risk was observed even 20–29 years later in a recent study [1], [2], [3], [19]. A cross-sectional study in Japan reported that former smokers who used to smoke heavily and who stopped smoking four or fewer years ago weighed more than never smokers [20], and that might have an influence on the association. However in our analysis, the increased risk among former smokers who quit smoking within five years was not altered even after controlling for BMI.

While limited but consistent epidemiological findings have indicated that smoking cessation increases the risk of diabetes for a certain period of time, the detailed mechanisms responsible for this association remain debatable. The increase in energy intake and the decrease in the resting metabolic rate that reportedly occur following smoking cessation [21], [22], might counter the beneficial effects of giving up smoking. Alternatively, smoking cessation may cause insulin resistance. However, only a few epidemiological studies have evaluated the level of insulin resistance among former smokers comparing current and never smokers with adjustments for potential confounders, and no study reported increased level of insulin resistance among former smokers comparing to current smokers [23], [24], [25]. Another possibility is that cigarette smoke has an adverse effect on β-cell function. The influence of cigarette smoke may persist, and the post-cessation effect on pancreatic function might differ from the effects of ongoing smoking. Our results partially favor this explanation with an additional implication of the greater effects on former smokers who smoked heavily or in those with major risk factors for diabetes. However, limited number of epidemiological studies has been conducted to evaluate the influence of smoking on β-cell function. A study reported the decreased β-cell function among former smokers comparing with current smokers [24], but not in the other study [26].

The main potential concern of the current study is that both the smoking status (exposure) and the development of diabetes (outcome) were self-reported. Typical pre-diagnosed symptoms of diabetes do not pose an immediate health threat [27], and hence, diabetes may often go undiagnosed. However, such misclassification in prospective studies would have biased the true association toward a null result when it occurs randomly. Weight was also self-reported. A previous study in Japan showed that men affected by diabetes were more likely to overestimate their weight [28]. If the bias in the self-reported weighted was related to the diabetic state in the same direction as this study, the misclassification was not likely to affect the study result, as the 5-year weight increase was not implied to be associated with increased risk of diabetes. However this may be uncertain because in a study in a Scottish population, the diabetic state among men was associated with a bias in self-reported weight in the opposite direction [29]. The self-reported BMI was lower than the measured BMI in the previous study of the JPHC cohort, especially among women, but the differences of the means were only 0.09 kg/m^2^ among men and 0.21 kg/m^2^ among women [7].

Another limitation is that non-respondents could have differed from respondents on characteristics, and that could have weakened the generalizability of the study results. Both among men and among women, subjects who were enrolled at the baseline but who did not answer either at the 5-year survey or 10-year survey were more likely to be younger on average than participants. The differential response according to smoking status was observed especially among women (5.0% of followed participants was current smokers at the baseline whereas 13.2% of those who did not answer the 5-year or 10-year survey was current smokers at the baseline). The impact of the non-respondents on the study results is unknown but the withdrawal of smoking women may have decreased the statistical power of the study. Furthermore, residual confounders may exist in our analysis, although we controlled for major risk factors of diabetes including obesity, physical activity and family history of diabetes.

The JPHC cohort was conducted using a large sample from the general population, and this study has several strong points. The study has a prospective design. The cessation of cigarette smoking as the main exposure variable was also prospectively noted, and that minimizes the possibility of a recall bias.

In summary, the current study indicated that men and women who newly quit smoking had an increased risk of developing diabetes. The five-year weight gain was not associated with an increased risk of diabetes among men, and even among new quitters, although several previous studies have speculated that weight gain following smoking cessation may have been a part of the underlying mechanism. New quitters with major risk factors for diabetes or who used to be heavy smokers were more likely to have a higher risk of developing diabetes among men. The adverse effects of smoking on health have been reported in numerous studies, and it is clear that smokers are strongly recommended to quit smoking. People attempting to cease smoking and medical professionals who are helping them should not neglect the possible elevation in the risk of diabetes.
