# Correction: Smoking Cessation Increases Short-Term Risk of Type 2 Diabetes Irrespective of Weight Gain: The Japan Public Health Center-Based Prospective Study

**DOI:** 10.1371/annotation/23aa7c42-9a4d-42a7-8f50-9d0ac4b85396

**Published:** 2013-04-15

**Authors:** Shino Oba, Mitsuhiko Noda, Kayo Waki, Akiko Nanri, Masayuki Kato, Yoshihiko Takahashi, Kalpana Poudel-Tandukar, Yumi Matsushita, Manami Inoue, Tetsuya Mizoue, Shoichiro Tsugane, for the Japan Public Health Center-based Prospective Study Group

We originally stated the risk of diabetes among newly cigarette quitters whose weight gain was less than 3 kg increased significantly compared with the equivalent counterparts among never smokers. However the association

was not statistically significant. We confirmed it by conducting the analysis with increasing the decimal precision. The odds ratio was 1.462 with 95 % confidence interval ranged from 0.998 to 2.144. 

